# Resolved and unresolved bioethical authenticity problems

**DOI:** 10.1007/s40592-020-00108-y

**Published:** 2020-04-07

**Authors:** Jesper Ahlin Marceta

**Affiliations:** Division of Philosophy, KTH Royal Institute of Technology, Stockholm, Sweden

**Keywords:** Authenticity, Autonomy, Biomedicine, Decision-making, Paternalism

## Abstract

Respect for autonomy is a central moral principle in bioethics. It is sometimes argued that authenticity, i.e., being “real,” “genuine,” “true to oneself,” or similar, is crucial to a person’s autonomy. Patients sometimes make what appears to be inauthentic decisions, such as when (decision-competent) anorexia nervosa patients refuse treatment to avoid gaining weight, despite that the risk of harm is very high. If such decisions are inauthentic, and therefore non-autonomous, it may be the case they should be overridden for paternalist reasons. However, it is not clear what justifies the judgment that someone or something is inauthentic. This article discusses one recent theory of what justifies judgments of inauthenticity. It is argued that the theory is seriously limited, as it only provides guidance in three out of nine identified cases. There are at least six authenticity-related problems to be solved, and autonomy theorists thus have reason to engage with the topic of authenticity in practical biomedicine.

## Introduction

“I wasn’t really bothered about dying, as long as I died thin.” The citation is an excerpt from an interview conducted with a person who talks about her anorexia nervosa (Tan et al. 2006, p. 274). The person reports that being thin was more important to her than being alive. Was her wish authentic? Was it really *hers*, in a substantial sense? The question has engaged bioethicists and medical practitioners, partly because the answer to it may also be important to another question, namely whether the person’s healthcare decisions should have been overridden.

One recent theory, namely Ahlin Marceta (2019), has been developed with the purpose of providing real guidance in such problems in practical biomedical settings. In this article, I argue that while that theory may be fruitful in cases related to decision-making, there are at least six authenticity-related problems that the theory from Ahlin Marceta (2019) cannot solve. Therefore, bioethicists have reason to engage in authenticity-related problems just as they have previously engaged in problems relating to decision-making capacity, voluntariness, and so on.

The article is structured as follows. First, I elaborate on the notion of authenticity and how it is related to personal autonomy and bioethics. Thereafter, I present the theory from Ahlin Marceta (2019) and discuss three cases in which the theory manages to provide normative guidance. The remainder of the article is devoted to six cases in which the theory fails to provide normative guidance. A brief final section concludes.

## Authenticity in biomedicine

### Autonomy and authenticity

To be autonomous is to be self-governed (Christman 2015). Respect for autonomy is one of the main moral principles in contemporary bioethics (cf. Beauchamp and Childress 2013). In concern for patients’ autonomy, bioethicists invoke concepts such as decision-making capacity (Grisso et al. 1997) and voluntariness (Nelson et al. 2011). That is, if a patient is not capable of making healthcare decisions, or if she is not making healthcare decisions independently from undue influences such as social or economic pressure, this has a negative effect on the degree of autonomy of her healthcare decisions. Non-autonomous healthcare decisions may sometimes be overridden for paternalist reasons, i.e., to protect the patient’s welfare interests.

During the twentieth century, informed consent practices have been incorporated in healthcare in large parts of the Western world with the aim of respecting and promoting patient autonomy (Jonsen 2000; Faden and Beauchamp 1986). In recent years, various bioethicists have raised the possibility of incorporating authenticity in autonomy-based practices in healthcare (Ahlin Marceta 2018a, b; Sjöstrand and Juth 2014; Villafranca 2019; White 2018; Zürcher et al. 2019). It is not entirely clear how the notion of authenticity should be conceptualized, although the term is usually understood to mean “genuine,” “real,” “true to oneself,” or similar.

The bioethicists’ concern has been that healthcare decisions must be authentic to be fully autonomous. Among the problems associated with this concern is that authenticity is difficult to detect in others (Ahlin 2018a; Sjöstrand and Juth 2014). More specifically, it is difficult to justify the judgment that someone else’s decision is inauthentic (Ahlin 2018b).

The perhaps most prominent tradition of thinking about authenticity has its roots in a series of books and articles from the 1970′s and 1980′s, of which Frankfurt (1971) and Dworkin (1988) may be the most noteworthy. In this tradition the authenticity of an act, decision, or desire is contingent on the agent’s attitude toward it. For illustration, consider a drug addict who has two conflicting wishes on two different desire-levels. On one level, she wants to take heroin. On a higher level, she wants to lead a long and healthy life. The desires are conflicting, and because of that conflict the desire on the lower level is deemed inauthentic. One criticism of so-called split-level (Friedman 1986) theories of authenticity is that desires on the higher level must also be endorsed on a yet higher level to be authentic, and desires on that level must also be endorsed on a yet higher level, and so on in an infinite regress (cf. Christman 2009, pp. 161–162; Taylor 2005). If the critics are right, there is something inherently problematic with the kind of authenticity theories which have gained most attention from philosophers and bioethicists in recent decades.

Other theories of authenticity include, for instance, those that put weight on the causal history of desires and those that focus on the coherence of full desire-sets. Jon Elster’s theory is one example of the former. In it, desires are inauthentic if they are “shaped by irrelevant causal factors, by a blind psychic causality operating ‘behind the back’ of the person” (Elster 1983, p. 16). In this line of thought authentic desires have a certain kind of origin, most often in some cognitive process of the desire-holder (Ahlin 2018a, p. 46). One example of a coherence-oriented theory is found in Bruce L. Miller, who writes that authentic actions are “consistent with the person’s attitudes, values, dispositions, and life plans” (Miller 1981, p. 24). In this line of thought actions, decisions, or desires are instead authentic if they are coherent with the desire-holder’s full set of desires (Ahlin 2018a, pp. 46–7).

### One practical theory of authenticity

In Ahlin Marceta (2019), a theory is developed aiming to provide guidance in practical authenticity-related problems. The theory is not concerned with the distinction between authenticity and inauthenticity, but with the distinction between justified and non-justified judgments of inauthenticity in others. Among other things, this entails that there can be inauthentic decisions that other agents—mainly for epistemic reasons—are not justified in judging as inauthentic. Therefore, the theory is focused on empirical factors that indicate inauthenticity and how agents should take them into consideration when making judgments of inauthenticity in others.

The theory is delimited to concern “persons whose medical condition may influence their decision-making so that they hurt themselves or others” (Ahlin Marceta 2019, p. 390). For such persons, and their possibly harmful healthcare decisions, “it is justified to judge that an underlying desire is inauthentic to the extent that it is due to causal factors that are alien to the person and to the extent that it deviates from the person’s practical identity” (p. 391).

In the theory, which builds from the idea of self-endorsement familiar from Frankfurt and Dworkin, two factors must be present for a judgment of inauthenticity to be justified (ibid):

**The factor of deviation**: It is a factor indicating inauthenticity that the desire under scrutiny does not cohere with how the desire-holder’s identity has developed over time and is presently being sustained.

**The factor of alien causes**: It is a factor indicating inauthenticity that the desire under scrutiny is due to causes that are not normal to how the desire-holder is otherwise construed, taking both physical and mental dispositions into consideration.

Both factors are expressed in degrees rather than in necessary and sufficient conditions, and are sensitive to judgment. It is, for instance, not stated a priori what it means for a cause to be “not normal” to how the desire-holder is otherwise construed. The theory requires practical and context-sensitive deliberation in particular cases.

Its application is a two-step process. First, it must be determined whether the person whose healthcare decisions are evaluated suffers from a medical condition that may influence their decision-making so that they are harmful to themselves or others. Second, it must be determined whether the two factors are present, and if so, to what extent. In Ahlin Marceta (2019), the process is demonstrated on a hypothetical (but empirically grounded) case of anorexia nervosa.

The theory is useful in at least three types of authenticity-related problems. The first concerns psychologically caused inauthenticity. Anorexia nervosa is usually treated as a psychiatric disorder. However, it should be noted that patients suffering from it can be fully competent to make healthcare decisions. Many can understand information relevant to their condition and the recommended treatment, reason about the potential risks and benefits of their choices, appreciate the nature of their situation and the consequences of their choices, and so on. Yet, they assess their own bodies, i.e., mainly their weight and physical appearance, unreasonably. Consider this excerpt from an interview conducted with an anorexia nervosa patient. It is representative also of other interviews in the same article (Tan et al. 2006, p. 274):

*Interviewer*: What is the importance of your weight and body size to you? “I just want to be thin.” *Interviewer*: How important is that to you? “Very.” *Interviewer*: Why? “It just is, it’s all I want.”

Thus, some anorexia nervosa patients have wishes that appear to be defective in some way, not as a matter of incompetence but of values. It is a problem to determine on what grounds these wishes are defective, and one suggestion is that it is because they are inauthentic.

Many would make the intuitively sound claim that the patient has inauthentic wishes because she has anorexia nervosa. However, inauthenticity is not listed among the diagnostic criteria for the disorder (see, e.g., American Psychiatric Association 2013). Therefore, although the patient’s wishes may be inauthentic, it is not because she has anorexia nervosa but for some reason external to the disorder. The intuitively sound claim that the patient’s wishes are inauthentic because she is anorexic is thus not empirically or conceptually valid. It could reasonably be argued that inauthenticity *should* be among the diagnostic criteria of anorexia nervosa, although it then remains to explain precisely what it is for something or someone to be inauthentic.

It may also be argued that our intuitions are misguided or misinterpreted in this case. They are not intuitions about the possible inauthenticity of the patient’s wishes, but about the patient’s welfare. That is, the intuition is in fact that the patient’s wishes are defective because it is not good to have them. Obviously, this can be true for some readers. Yet, various clinicians and bioethicists, such as, e.g., Hope et al. (2011), Sjöstrand and Juth (2014), and Tan et al. (2006), have expressed and analyzed the possible problem of anorexia nervosa patients’ wishes in terms of authenticity. Their analyses do not appear to rest on misguided or misinterpreted intuitions, but on the stable and considered view that there is some authenticity-related problem with such wishes.

The target case in Ahlin Marceta (2019) is precisely a case of anorexia nervosa, and I will not repeat the analysis here. It should be sufficient to declare that the theory is (arguably) fruitful in cases where there appears to be problems connected to psychological causes and wishes that are intertwined with the diagnostic criteria of some disorder.

The second type of case that the theory is fruitful in concerns physically caused inauthenticity. In a case study, Burns and Swerdlow (2003) report of an otherwise normal 40-year old man who suddenly developed a sexual interest in children. The man had no previous pedophilic symptoms and did not want to have them either; among other things, he underwent a 12-step program for sexual addiction to be able to lead a normal life. Upon medical examination, it was found that the man’s sexual desires were due to a brain tumor. He had developed a right orbitofrontal tumor which affected him cognitively and behaviorally. When the tumor was removed, the pedophilic symptoms disappeared. When the symptoms later returned, it was found that that the tumor had done so too. Thus, there is a clear and unambiguous causal relationship between the man’s brain tumor and his sexual desires. Or, that is at least how the case is treated in Ahlin (2018b, p. 369), where the theory from Ahlin Marceta (2019) first began to take shape. There seems to be authenticity-related problems connected to the case.

The man’s medical condition could have influenced his decision-making negatively in the sense described in Ahlin Marceta (2019). This is obvious from the case description. Furthermore, both the factor of deviation and the factor of alien causes are present, although this brief case description does not state to what extent. Nonetheless, it should be reasonably clear that the theory in Ahlin Marceta (2019) is applicable in cases where desires are due to physical causes, although its full potential can only be realized in more detailed particular instances.

The third type of case concerns surprising wishes. Consider the hypothetical case of Anna, “a young and promising professional ballet dancer” (Ahlin 2018a, p. 44). Anna loves her work, has moved across the nation to attend the best ballet schools, set aside personal relationships that conflicted with her career, and is known by those who are close to her to love dancing more than anything else. In the case, Anna has suffered a serious leg injury and must undergo minor surgery to avoid complications that will in time make amputation necessary. Anna is competent to make healthcare decisions and is fully informed about the consequences of her decisions, yet she refuses to undergo surgery. Her treating clinician reflects upon the case and believes that Anna’s decision rests on inauthentic desires.

The case is intended to illustrate that it is often surprises that bring attention to the notion of authenticity; as long as people make decisions that are not unexpected, we do not consider them in terms of authenticity. But, with support from Ahlin Marceta (2019), the case also shows that decisions are not inauthentic merely because they are surprising, not even if the decisions are surprising to the extent that they conflict with everything that is known about the decision-maker. Judgments of inauthenticity require a real and elaborate explanation. In the case of Anna, the causal history of her desires is unknown and therefore the requirement to meet the factor of alien causes is not fulfilled. Thus, the theory in Ahlin Marceta (2019) provides guidance here.

The three cases share one feature, namely that they concern *decision-making* and *desires*. The theory from Ahlin Marceta (2019), which is developed for precisely such problems, can therefore be fruitfully applied to them. However, not all authenticity-related problems concern decision-making and/or desires. Therefore, in all other cases the theory from Ahlin Marceta (2019) is fruitless.

There are alternative theories of authenticity that do not concern decision-making or desires. For instance, Katharina Bauer (2017) offers an alternative approach, namely the focus on what it is to be an authentic *person*. The ideal of being an authentic person, in Bauer’s proposal, is a combination of the ideal of expressing and unfolding one’s individual personality and the ideal of being an autonomous person who is morally responsible (p. 579). In more elaborate terms, the ideal is comprised of (1) aspects of being authentic by being a self with distinctive characteristics of an individual personality. These aspects include the free unfolding of one’s individual personality, expression of oneself in acting and living, and being true to one’s own convictions, beliefs, ideals, life-plans, and projects (ibid). Furthermore, the ideal is comprised of (2) aspects of being authentic by being “a person” in terms of an autonomous (moral) agent. These aspects include giving reasons and taking moral responsibility for one’s actions, being a reflective “self-evaluator,” and being a trustworthy partner of social interaction (ibid). One other alternative is to instead focus on what it is to lead an authentic *life* (cf. Taylor 1991).

These alternatives have gained less attention from bioethicists than the decision-oriented approach—perhaps because bioethicists’ main focus is on autonomous decision-making. However, because there are at least six authenticity-related problems that cannot be solved using the theory from Ahlin Marceta (2019), or so it will be argued, it may be worthwhile to explore whether the alternative approaches can be further developed to justify practical judgments of authenticity.

## Six authenticity-related cases

The six authenticity-related bioethical cases that the theory from Ahlin Marceta (2019) cannot solve are: (1) Unstable desire-sets, (2) lack of desires, (3) medically induced authenticity, (4) inauthentic recovery, (5) indoctrinated desires, and (6) false selves. I will go through them in that order and discuss what solving the problems requires from theorists.

### Case 1: unstable desire-sets

Among other things, patients suffering from borderline personality disorder (BPD) are characterized by unstable “selves,” which has prompted ethicists to consider the ethics of caring for BPD patients in terms of authenticity (Lester 2009). A BPD patient could, for instance, display sudden and dramatic shifts in goals, values, vocational aspirations, types of friends, and so on (ibid, p. 284). In extreme situations, BPD patients can make a series of mutually incompatible healthcare decisions resting on unstable desires. For instance, a BPD patient may request forced medication, as only that enables her to go through psychotherapy, and minutes later refuse medication, as one of its side effects is that it clouds her thinking. Healthcare personnel cannot adhere to both wishes.

The main authenticity-related problem in this case appears to be that BPD patients have desire-sets that are too unstable. Surely, a normal person could have authentic but conflicting wishes in subjects of minor importance, such as an authentic wish to eat ice cream and an authentic wish to not eat sugar. Also, normal persons could reasonably be authentically indecisive, at least to some extent. But BPD patients are unstable in a way, and to an extent, that seems to call for judgments of inauthenticity. That is, there is a seriousness to their symptoms that makes it reasonable to assess their personality, or their decisions, in terms of authenticity. However, it remains for theorists to explain precisely why and how their instability is an authenticity-related problem, if at all.

The theory in Ahlin Marceta (2019) is incapable of treating the main moral problem in this case. The theory could be applied to particular decisions made by BPD patients, although the problem is not the decisions per se but that they rest on unstable desire-sets and that this is a possible state in inauthenticity. Therefore, provided that this instability is an authenticity-related problem, some other theory than that from Ahlin Marceta (2019) should be developed to treat it.

### Case 2: lack of desires

The late stages of schizophrenia may include “negative” symptoms such as underactivity, blunting of affect, passivity, and lack of initiative (American Psychiatric Association 2013). Schizophrenics in this stage can sometimes lead reasonably normal lives, while being completely indifferent to anything that happens to them and how their lives go. It does not matter to them whether they are healthy, live in a comfortable home, or have meaningful relationships with others. They can be described as living without any wishes.^1^

The question can be raised whether this condition is authentic, i.e., whether a person can authentically lack wishes. In some cases a state of mind that is free of wishes is desirable, such as when it is the wanted result from deliberate meditation. Buddhists, mindfulness practitioners, and others, seek to not have any desires. However, it is different to be in that condition due to some medical disorder. Thus, it is a problem for authenticity theorists to clarify whether it is possible to authentically lack wishes, where this lack is due to some disorder, and if so, also why.

Furthermore, when these questions have been resolved, a theory must be developed that can be applied to reliably determine whether a desire-free condition or state of mind is inauthentic. As the problem here is not to determine whether any particular decision rests on inauthentic desires, the theory from Ahlin Marceta (2019) cannot be applied for guidance.

### Case 3: medically induced authenticity

In the first chapter of his book *Listening to Prozac* (1993), Kramer reports of Tess, a patient whose personal story is extraordinary. Among many other things, Tess was a victim of child abuse. She suffered from depression and had suicidal thoughts. After various failed attempts at medication and therapy Kramer prescribed Prozac, which at the time had recently been released by the U.S. Food and Drug Administration. Soon thereafter, Tess showed a remarkable change. Her work became more satisfying, her social relationships changed to the better, and she was “astonished at the sensation of being free from depression” (p. 7). After nine months, Tess went off medication and continued doing well. About eight months after that, she told Kramer that she was slipping. She said, “I’m not myself” (p. 10). Thus, Prozac made Tess authentic (per self-report).

The case draws out a conflict of intuitions. On the one hand, it is intuitive to hold that Tess’s self-reports of authenticity are real simply because they are self-reported. On the other, it is counterintuitive to hold that she is authentic, as it is known that her condition is induced by medication. The theory from Ahlin Marceta (2019) cannot resolve these conflicts, as they do not concern decision-making or desires. There is thus reason for authenticity theorists to organize and explain these conflicting intuitions in new theoretical work.

One possible explanation of the case is that Prozac helped Tess to “find” the authentic self that she was before she was abused as a child (provided that the abuse caused the inauthenticity). However, this explanation is more complex than what first appears. It rests on the assumption that authenticity is about something that does not change over time, namely some personhood-related entity which remains the same in both Tess pre-abuse and in Tess post-abuse. Thereby, it commits to theories of personhood, philosophy of mind, and possibly also phenomenology, according to which a person is something intertemporally fixed. These theories are not obviously true. Thus, the explanation is simple and attractive at first glance, but upon closer examination it becomes clear that it carries a large theoretical load that makes it very complex.

One other possible explanation is that Tess confuses who she *is* with who she *wants to be*. She wants to be the person that Prozac helps her to be, and therefore she states that this person is who she really is. This explanation is also more complex than what first appears. If it is correct, normally informed and competent persons can be mistaken about who they really are, in terms of authenticity. The explanation may disqualify theories of authenticity that are oriented around self-endorsement, which have otherwise been prominent in authenticity theorizing since Frankfurt (1971) and Dworkin (1988).

In conclusion, intuitively reasonable explanations of the case with Tess are theory-dependent and complex upon closer examination. It remains for authenticity theorists to treat cases of medically induced authenticity in greater detail.

### Case 4: inauthentic recovery

Some disorders can be treated with either medicine or psychotherapy (or both). It can be argued that, for reasons of authenticity, psychotherapy is a better option than medicine. This line of thought has been explored by, e.g., Kass (2003, pp. 22–3):

In most of our ordinary efforts at self-improvement, either by practice or training or study, we sense the relation between our doings and the resulting improvement, between the means used and the end sought. There is an experiential and intelligible connection between means and ends; we can see how confronting fearful things might eventually enable us to cope with our fears. We can see how curbing our appetites produces self-command. […] In contrast, biomedical interventions act directly on the human body and mind to bring about their effects on a subject who is not merely passive but who plays no role at all. […] The relations between the knowing subject and his activities, and between his activities and their fulfillments and pleasures, are disrupted.

It is one argument that psychotherapy is better than medicine because of some positive secondary effects, such as a strengthened self-esteem or longer lasting medical result. I am not concerned with that here. But, it can also be argued that psychotherapy is better than medicine because of some authenticity-related reason. That is, the opinion is reasonable that authentic recovery from disorder is better than inauthentic recovery. But, the opinion rests on the idea that there *is* such a thing as inauthentic recovery, and it is not immediately clear that there is theoretical support for this idea beyond mere intuition.

This is different from questions of whether someone’s decision between treatment and therapy is authentic. The problem for theorists, if it is a problem at all, is to make a clear and unambiguous distinction between authentic and inauthentic recovery processes.^2^ Obviously, Ahlin Marceta (2019) is not useful here.

### Case 5: indoctrinated desires

Consider this thought example (Taylor 2005, p. 11):

[Imagine] a child at time t whose mother wished him to learn to play the piano and who beat him if he did not practice. As time passes and the child grows more proficient at playing, he discovers (at time t1) that his mother’s belief that piano playing suited him was right, and he comes to love playing – even though he still repudiates the means by which his mother brought him to this position.

The thought example is intended to bring out a conflict of intuitions; intuitively, the boy’s love for playing the piano is formed in the wrong way and is therefore inauthentic, but the boy endorses his own love for playing the piano upon informed and critical self-reflection and therefore it is intuitive to hold that it is authentic. Different authenticity theories explain such cases of manipulation or indoctrination differently. Theories that emphasize the causal history of desires, such as, e.g., Elster’s (1983), would determine that the child’s love for playing the piano is inauthentic. Theories that focus on self-endorsement, such as, e.g., Frankfurt’s (1971) and Dworkin’s (1988), would instead determine that the child’s love for playing the piano is authentic.

One more straightforward example of indoctrination is discussed by Noggle (2005, p. 102):

*Edgar the Evil* is a son of a crime boss who rears him to follow in his footsteps. Using standard child-rearing techniques, he encourages Edgar’s more selfish and violent impulses and discourages empathy and compassion. As Edgar reaches adulthood, he is quite thoroughly evil.

The commonly shared intuition is that Edgar is not authentically evil. Edgar the Evil is analogous to people who, for instance, grow up in religious sects or live under oppressive patriarchic social conditions. Sometimes such people make dubious healthcare decisions that indicate inauthenticity. For instance, many bioethicists today agree that the wishes of a Jehovah’s Witness who refuses blood transfusion should be respected for anti-paternalist reasons. Further analysis may be appropriate concerning their possible inauthenticity; perhaps there are similar cases in which reliable indicators of inauthenticity provide sufficient grounds for paternalist interventions.

It remains for authenticity theorists to organize and explain the various conflicting intuitions in cases of manipulation or indoctrination, and to provide clear and unambiguous action-guidance with regard to them. The theory in Ahlin Marceta (2019) is partially guiding here, but it does not answer the relevant questions. Presumably, neither manipulation nor indoctrination are medical conditions. Therefore, manipulated or indoctrinated patients are not the kind of persons that, according to Ahlin Marceta (2019), are justifiably targeted by inauthenticity judgments. However, this normative guidance is not satisfying. It side-steps the relevant moral problem, namely the possible inauthenticity of decisions that are due to manipulation and indoctrination, rather than solves it.

### Case 6: false selves

Winnicott (2007) introduced a thought example called the “False Self” which has been used as a paradigm model of inauthentic behavior (see, e.g., Velleman 2002, pp. 97–8). In the example, we are to picture a person who “laughs at what he thinks he is supposed to find amusing, shows concern for what he thinks he is supposed to care about, and in general conforms himself to the demands and expectations of others” (Velleman 2002, p. 97). He fails to be motivated “from within his true self” and is therefore inauthentic (ibid). The lesson we are supposed to learn is that conformity, in some sense, negates authenticity. However, it is not obvious that the example succeeds in showing that. Taylor comments on the False Self person that, “while his laughter might not be authentic in the sense of its expressing genuine amusement, it would be authentic in the sense of being representative of this person’s other-directedness. It would be authentically inauthentic” (Taylor 2009, p. 32). In other words, the False Self person might be an authentically other-directed person.

Taylor does have a point, although there is something distressing about his remark. The False Self example draws attention to the intuition that there is something inauthentic about people who conform to what they believe to be others’ wishes rather than to formulate and follow their own. But the example is too strong. Humans are socially embedded beings; everyone conforms to others’ expectations to some extent, at least during periods of our lives. In many cases, we tend to think that people who fail to conform to others’ expectations lack social skills. We even hope that our children learn the social balance between following one’s own desires and conforming to others’. Thus, it is difficult to draw the straight and unambiguous line between “self-directedness” and “other-directedness” that the False Self is intended to elucidate. However, the thought of a person who is “authentically inauthentic,” as Taylor suggests, is as distressing as the thought of a person who is completely insensitive to the expectations of others. In reality, the normal case is likely that authentic people are somewhere in between fully self-directed and fully other-directed.

There is disagreement among authenticity theorists regarding problems that are connected to the tension between social influences and the self. It is possible that the main merit of the False Self example and Taylor’s comments is that they illuminate one problem associated with constructing a hypothetical ideal of authenticity; perhaps any ideal model of authenticity would be torn apart by the forces in the dialectics above. No person can be either authentically fully self-directed nor authentically fully other-directed, and therefore any ideal that is oriented around either extreme is inherently flawed. Instead, it may be argued, a theory of authenticity should be non-ideally constructed, and account for the tension between social influences and the self already from the outset.

The theory in Ahlin Marceta (2019) is non-ideal in this sense. However, the problem presently described is not of the kind that Ahlin Marceta (2019) can solve, as the theory is silent on the possibly authenticity-related problem of other-directedness.

## Conclusions

Authenticity issues relate to a number of different problems; some are related to decision-making, others rather concern personhood or being in some condition. Therefore, there is likely not one universal solution to authenticity-related problems, but various particular solutions.

As mentioned briefly in the discussion of case 6, it is possible that bioethicists should further consider a non-ideal methodological approach to authenticity-related problems. Most (or all) theories of authenticity are comprised of some hypothetical ideal of authenticity, in the sense that they are constructed of propositions such as “X is authentic if and only if Y.” Then, the theories suggest that practitioners should scrutinize X’s (i.e., desires, lives, persons, etc.) and observe whether and to what extent they have or are Y. It may instead be fruitful to follow Ahlin Marceta (2019) and adopt a non-ideal approach. Such approaches, which are sometimes also described as “realist,” “problem-oriented,” or “bottom-up,” may start from the case at hand rather than from some hypothetical model of authenticity and attempt to describe what is problematic about it in particular terms. Bioethicists should at least explore the possibility of taking a new methodological grip on authenticity-related problems.

To summarize, this article collects six authenticity-related cases in biomedicine that the theory from Ahlin Marceta (2019) cannot solve. It has been proposed that bioethicists should explore alternative methodological approaches to the notion of authenticity and its applications in biomedicine. There is yet a lot of analytical work to be done regarding authenticity in biomedical contexts. Bioethicists have reason to engage in authenticity theory precisely as they have previously engaged in theorizations of concepts such as decision-making capacity and voluntariness.

## Footnotes

1 I adopt this characterization from dialogues with psychiatrists.

2 See also Fredrik Svenaeus (Svenaeus 2009), who argues that there is no ethically relevant difference between psychopharmacological and psychological self-change.
